# Supplementary material for: Epithelial expression of Gata4 and Sox2 regulates specification of the squamous–columnar junction via MAPK/ERK signaling in mice
Source: Nat Commun. 2021 Jan 25;12:560. doi: 10.1038/s41467-021-20906-0 (PMC7835245; doi:10.1038/s41467-021-20906-0)
Supplement: Supplementary file 1 — Supplementary Information [file 41467_2021_20906_MOESM1_ESM.pdf]

Epithelial expression of *Gata4* and *Sox2* regulates specification of the squamous–columnar junction via MAPK/ERK signaling in mice

Sankoda et al.

Inventory of Supplementary Information

Supplementary Figures (Supplementary Figures 1-7)

Legends to Supplementary Figures

Supplementary Table (Supplementary Table 1-2)

Supplementary Figure 1

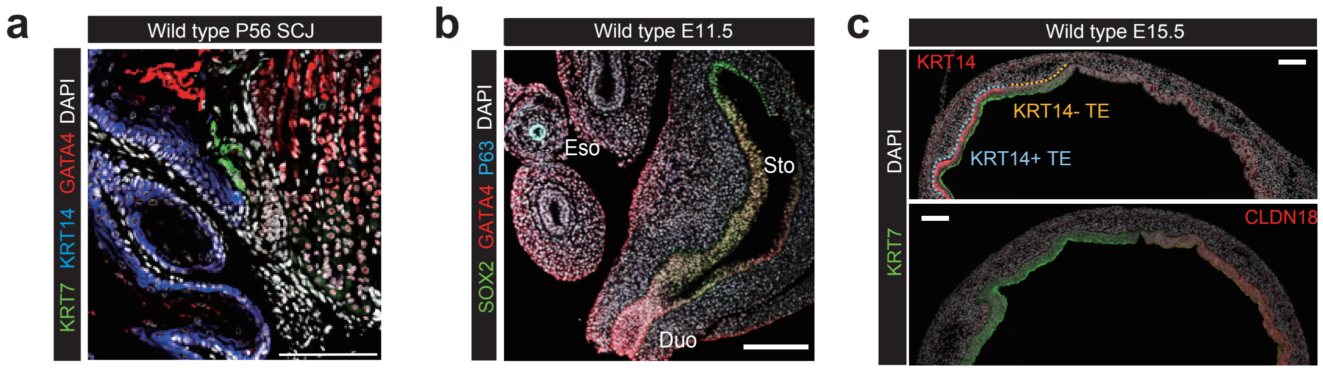

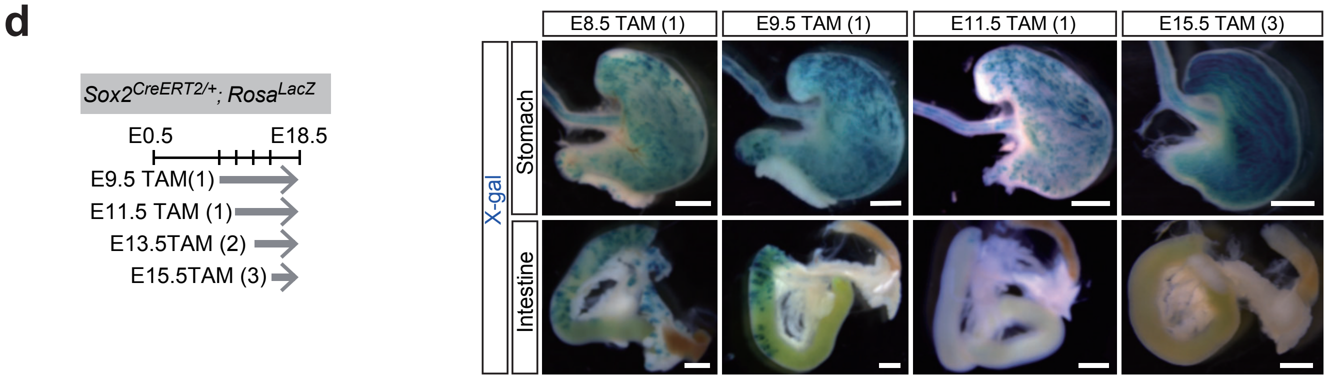

Day of TAM i.p. (dose)		E8.5 (1 mg)	E9.5 (1 mg)	E11.5 (1 mg)	E15.5 (3 mg)
The number of fetuses (pregnant females)		14 (7)	12 (5)	6 (3)	12 (3)
Contribution of *Sox2*-expressing cells	Esophagus	14/14	12/12	6/6	12/12
	PS	14/14	12/12	6/6	12/12
	DS	14/14	12/12	6/6	12/12
	Duodenum	14/14	12/12	6/6	0/12
	Pancreas	13/14	7/12	0/6	0/12
	Liver	0/14	0/12	0/6	0/12

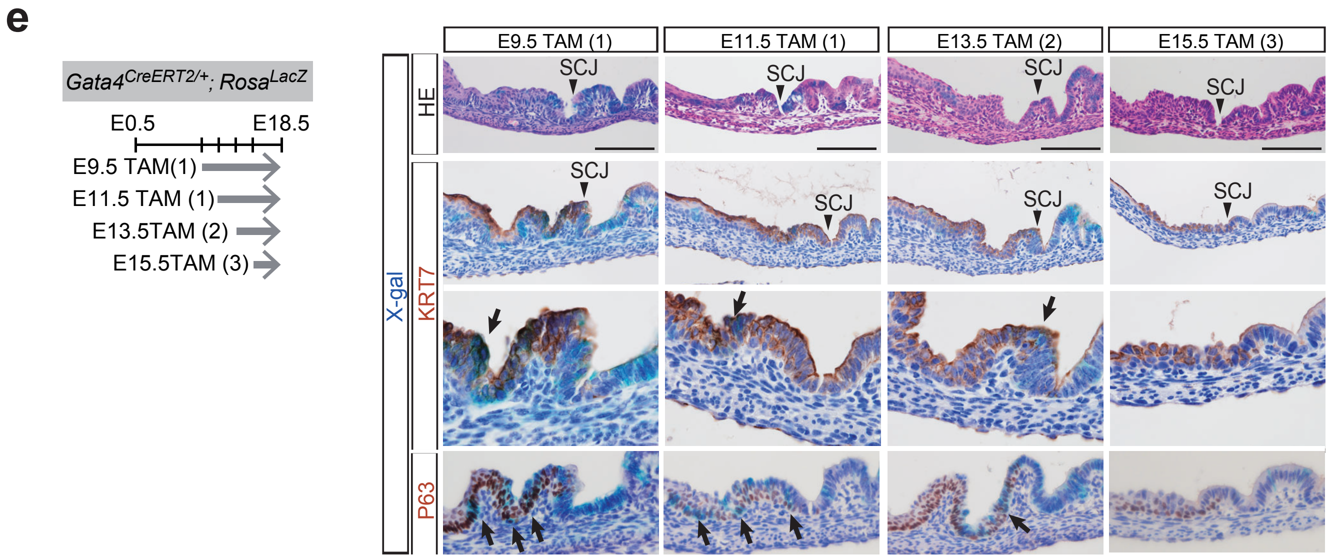

Supplementary Figure 1: Expression patters of SOX2 and GATA4 in KRT7^{+} transitional epithelium during development

(a) IF analyses of KRT7, KRT14, and Gata4 for SCJs of the stomachs at postnatal day 56. Presented data are a representative image of n=3. Scale bar, 100 μm.

(b) IF analyses of SOX2, GATA4, and P63 for esophagus, stomach, and duodenum of the wild-type embryos at E11.5. Eso, esophagus; Sto, stomach; Duo, duodenum. Presented data are a representative image of n=5. Scale bar, 100 μm.

(c) IF analyses of KRT7, KRT14, and CLDN18 for proximal stomachs at E15.5. Presented data are a representative image of n=3. Scale bar, 100 μm.

(d) Top: Whole-mount X-gal staining for stomach, duodenum, and pancreas of the *Sox2$^{CreERT2/+}$; Rosa^{lacZ}* embryos at E18.5. Scale bars, 1 mm. Bottom: A table summarizes the distributing ratios of *Sox2*-expressing cells (X-gal^{+}) to the organ tissues derived from foregut endoderm in *Sox2$^{CreERT2/+}$; Rosa^{lacZ}* embryos at E18.5. PS, proximal stomach; DS, distal stomach. *Sox2*-expressing cells from E8.5 to E15.5 contributes to the esophagus, proximal and distal stomach but not to the liver. Note the loss of differentiation potential to the pancreas and duodenum at later stages.

(e) H&E staining and IHC analyses of KRT7 and P63 for SCJs of the *Gata4$^{CreERT2/+}$; Rosa^{lacZ}* embryos at E18.5 after whole-mount X-gal staining. Arrows indicate the KRT7^{+} / Xgal^{+} or P63^{+} / X-gal^{+} epithelial cells in the proximal stomachs. Presented data are a representative image of n=4. Scale bar, 100 μm.

Supplementary Figure 2

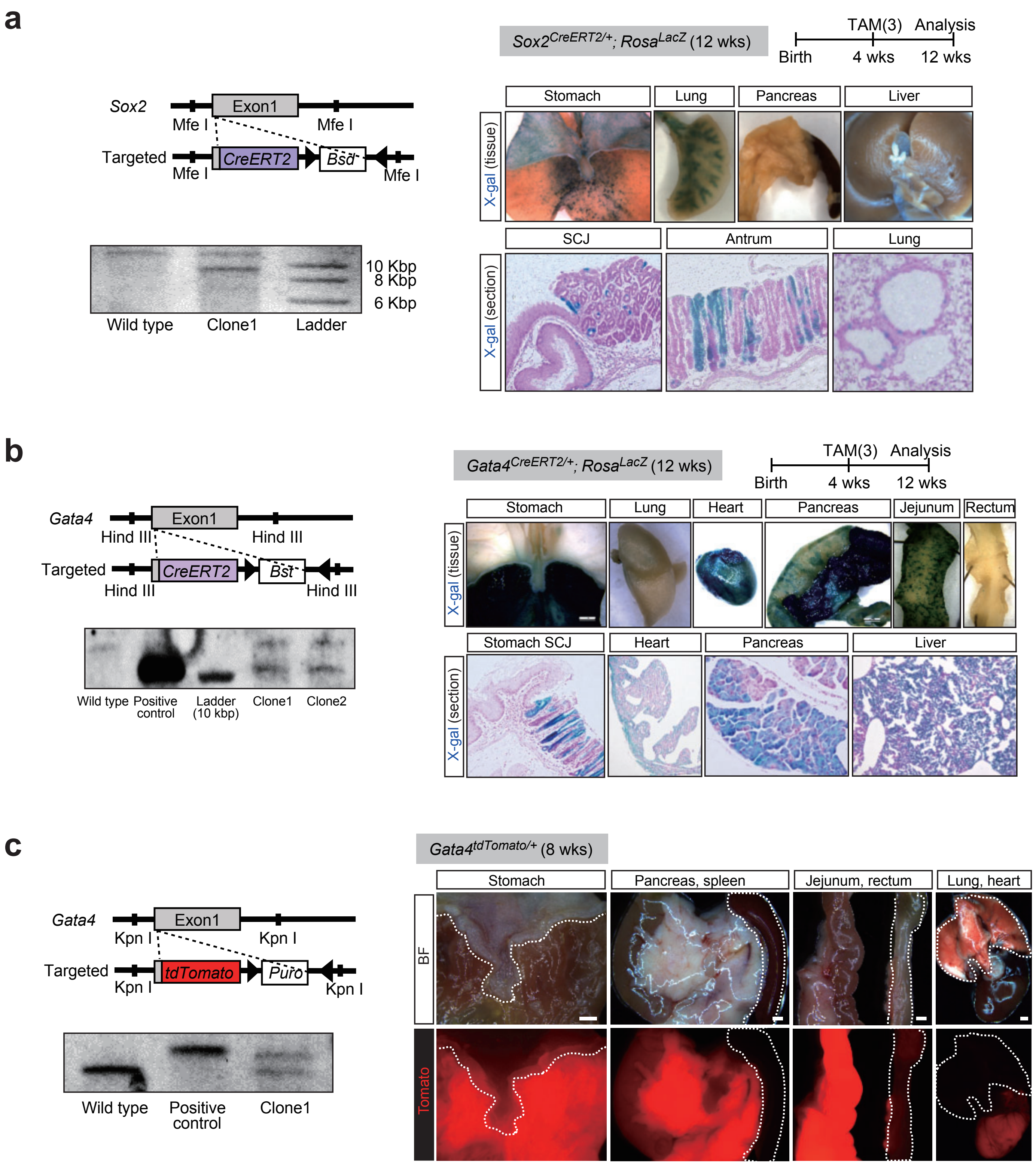

Supplementary Figure 2: Generation of the *Sox2*$^{CreERT2/+}$, *Gata4*$^{CreERT2/+}$, and *Gata4*$^{td\ Tomato/+}$ mice by homologous recombination

(a) Left: A schematic illustration of homologous recombination with a targeting vector containing *CreERT2* at endogenous *Sox2* locus. Southern blotting indicates successful recombination in ESCs. Right: Whole mount X-gal staining on various organs of *Sox2*$^{CreERT2/+}$*; Rosa*^{lacZ} adult mice and X-gal staining on the histological sections of the stomach and lung of *Sox2*$^{CreERT2/+}$*; Rosa*^{lacZ} mice. Recombination is observed in the antrum gland unit and tracheal epithelium. n=3. Scale bars, 100 µm.

(b) Left: A schematic illustration of homologous recombination with a targeting vector containing *CreERT2* at endogenous *Gata4* locus. Southern blotting indicates successful recombination in ESCs. Right: Whole mount X-gal staining on various organs of *Gata4*$^{CreERT2/+}$*; Rosa*^{lacZ} adult mice and X-gal staining on the histological sections of the stomach, heart, lung, pancreas, and liver of *Gata4*$^{CreERT2/+}$*; Rosa*^{lacZ} mice. Recombination is observed in the corpus gland unit, cardiomyocytes, pancreatic acinar cells, and hepatocytes. n=3. Scale bars, 100 µm.

(c) Left: A schematic illustration of homologous recombination with a targeting vector containing *td Tomato* at endogenous *Gata4* locus. Southern blotting indicates successful recombination in ESCs. Right: Fluorescent images of various organs of *Gata4*$^{td\ Tomato/+}$ adult mice. td Tomato fluorescence is observed in the glandular stomach, pancreas, duodenum, jejunum, and heart. n=3. Scale bars, 1 mm.

Supplementary Figure 3

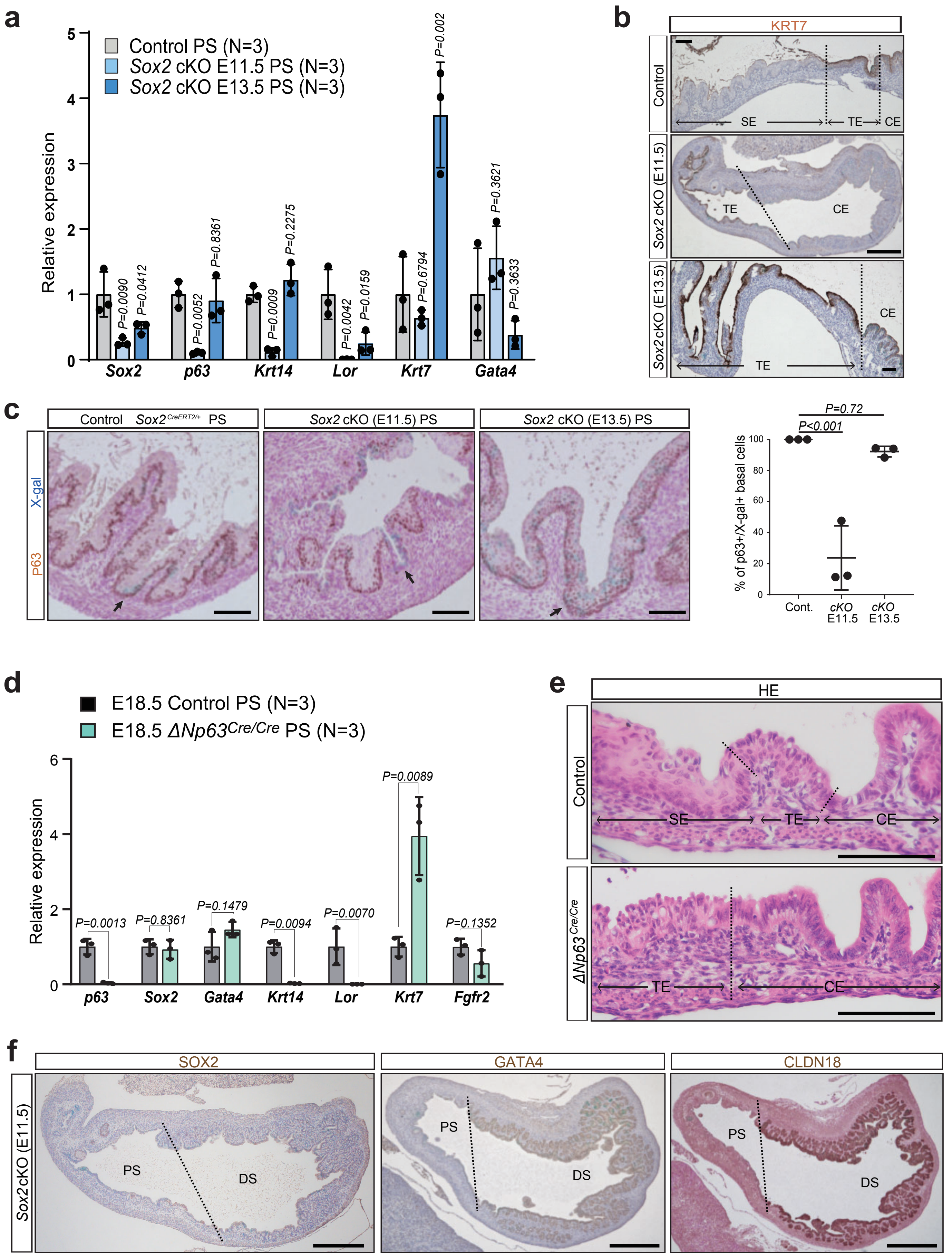

Supplementary Figure 3; Roles of SOX2 and P63 in the specification of KRT7$^+$ transitional epithelium into squamous epithelium

(a) Relative expressions of indicated genes in the control, *Sox2 cKO (E11.5)*, and *Sox2 cKO (E13.5)* proximal stomachs at E18.*5*. The CT values of each gene are normalized by *B2M*. The average ΔCT values for control proximal stomachs are set to 1. n=3 independent experiments. Data are presented as mean values ± SD. one-way ANOVA.

(b) IHC analyses of KRT7 for the stomachs of control, *Sox2 cKO (E11.5)*, and *Sox2 cKO (E13.5)* embryos at E18.5. Presented data are a representative image of n=3. Scale bar, 100µm.

(c) Left: IHC analyses of P63 for the proximal stomachs of control, *Sox2 cKO (E11.5)*, and *Sox2 cKO (E13.5)* embryos after the whole mount X-gal staining. n=3. Scale bar, 100µm. Arrows indicate the X-gal$^+$ cells. Right: quantification of the number of P63$^+$ cells in the X-gal$^+$ basal cells in the proximal epithelial cells. Data are presented as mean values ± SD. n = 3 independent experiments. one-way ANOVA.

(d) Relative expressions of indicated genes in the control and *ΔNp63 KO* proximal stomachs at E18.5. The CT values of each gene are normalized by *B2M*. The average ΔCT values for the control proximal stomachs are set to 1. n=3 independent experiments. Data are presented as mean values ± SD. two-sided *t*-test.

(e) H&E staining for the control and *ΔNp63 KO* junctions at E18.5. Presented data are a representative image of n=4. Scale bar, 100µm.

(f) IHC analyses of SOX2, GATA4 and CLDN18 on the stomachs of the *Sox2 cKO (E11.5)* embryo. Presented data are a representative image of n=3. Scale bar, 100µm.

Supplementary Figure 4

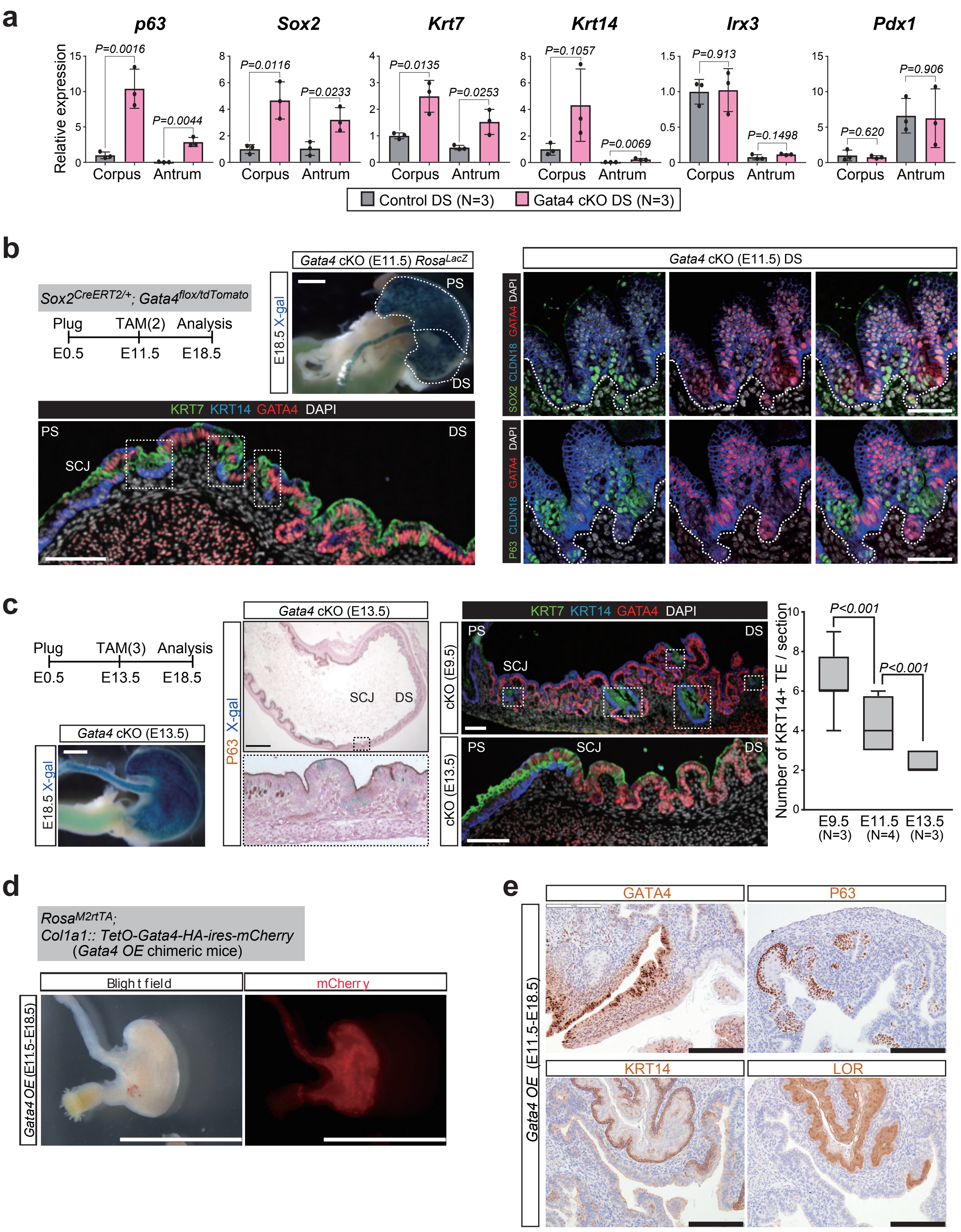

Supplementary Figure 4: Epithelial GATA4 specifies KRT7$^+$ transitional epithelium into columnar epithelium by acting against SOX2

(a) Relative expressions of the indicated genes in control and *Gata4 cKO (E9.5)* distal stomachs. Corpus and antrum were manually separated. The CT values of each genes are normalized by *B2M*. The average ΔCT values for control corpus are set to 1. n=3 independent experiments. Data are presented as mean values ± SD. two-sided *t*-test.

(b) IF analyses for the *Gata4 cKO (E11.5)* distal stomachs at E18.5. *Gata4*-ablation at E11.5 resulted in the ectopic emergence of the KRT14$^+$KRT7$^+$ transitional epithelium in the distal stomach. Presented data are a representative image of n=4.

(c) Analyses for the *Gata4 cKO (E13.5)* distal stomachs at E18.5. Left & Middle: *Gata4*-ablation at E13.5 did not cause the ectopic emergence of the P63$^+$ cells or KRT14$^+$KRT7$^+$ transitional epithelium in the distal stomach. Presented data are a representative image of n=3. Right: box-and-whisker plot showed the number of the solitary transitional epithelium in the histological sections of *Gata4 cKO (E9.5)* (n=3), *Gata4 cKO (E11.5)* (n=4), and *Gata4 cKO (E13.5)* (n=3) stomachs. Solid lines in each box indicate the median. Bottom and top of the box are lower and upper quartiles, respectively. one-way ANOVA.

(d) Fluorescent images of the *Col::tetO-Gata4-HA-IRES-mCherry*; *Rosa^{rtTA}* chimeric embryos. Presented data are a representative image of n=4. Scale bar, 1mm.

(e) IHC analyses of GATA4, P63, KRT14, and LOR for the proximal stomachs of the *Gata4 OE* embryos at E18.5. Presented data are a representative image of n=4. Scale bar, 100µm.

Supplementary Figure 5

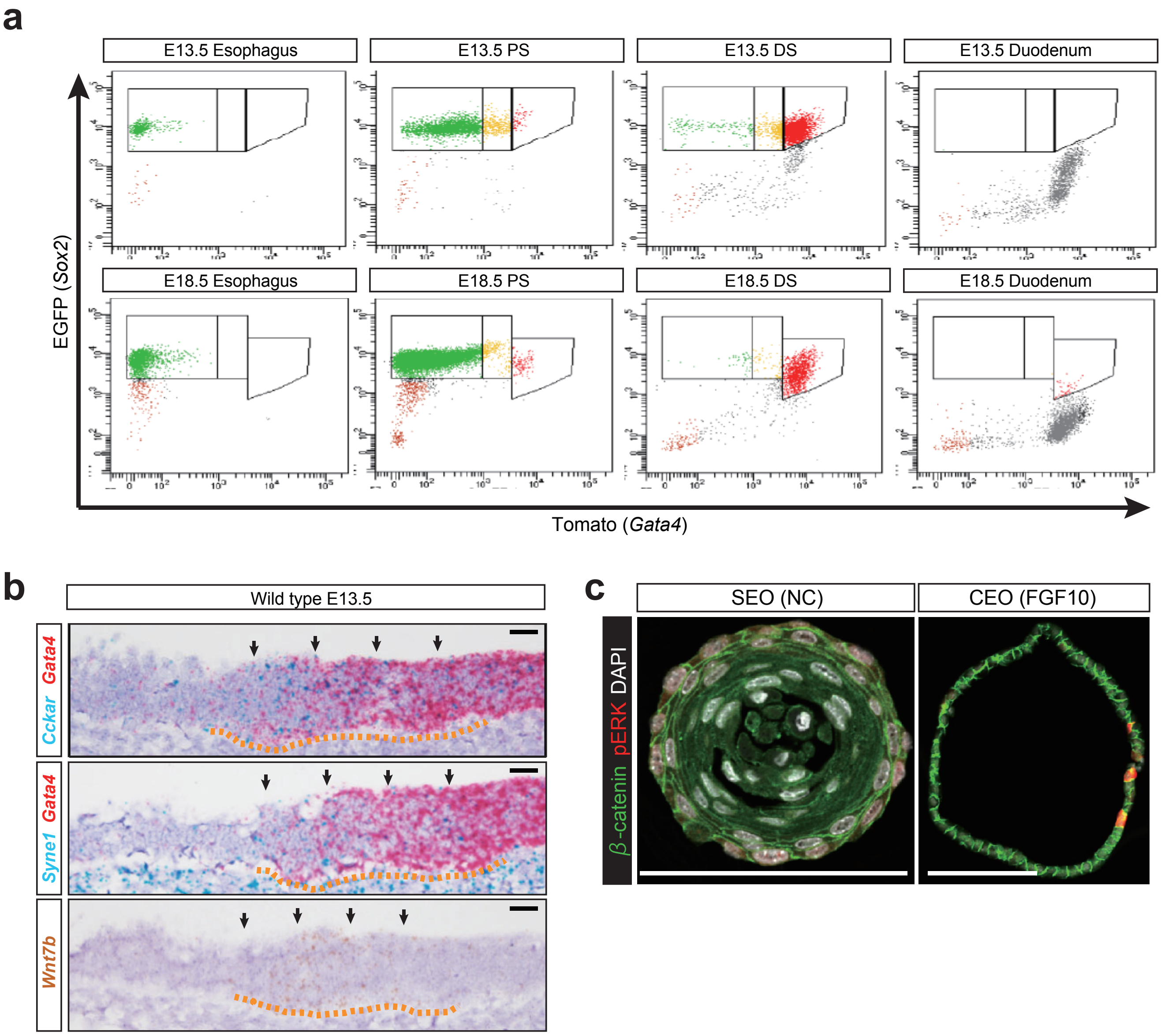

Supplementary Figure 5; *Sox2^{mid}Gata4^{mid}* cells localizing in between *Sox2^{hi}* and *Gata4^{hi}* cells have unique characteristics

(a) Gating strategies of FACS analyses for the *SGGT* stomachs at E13.5 and E18.5.

(b) RNA-ISH analyses of *Cckar*, *Syne1*, *Gata4*, and *Wnt7b* for wild-type stomach at E13.5. Top, middle and bottom panels are serial sections. Arrows indicate *Cckar*$^{+}$ *Wnt7b*$^{+}$ *Syne1*$^{+}$ cells located in the intermediate epithelium. Presented data are a representative image of n=3. Scale bar, 100 µm.

(c) IF analyses of pERK and β-catenin in SEOs and CEOs. Presented data are a representative image of n=3. Scale bar, 100 µm.

Supplementary Figure 6

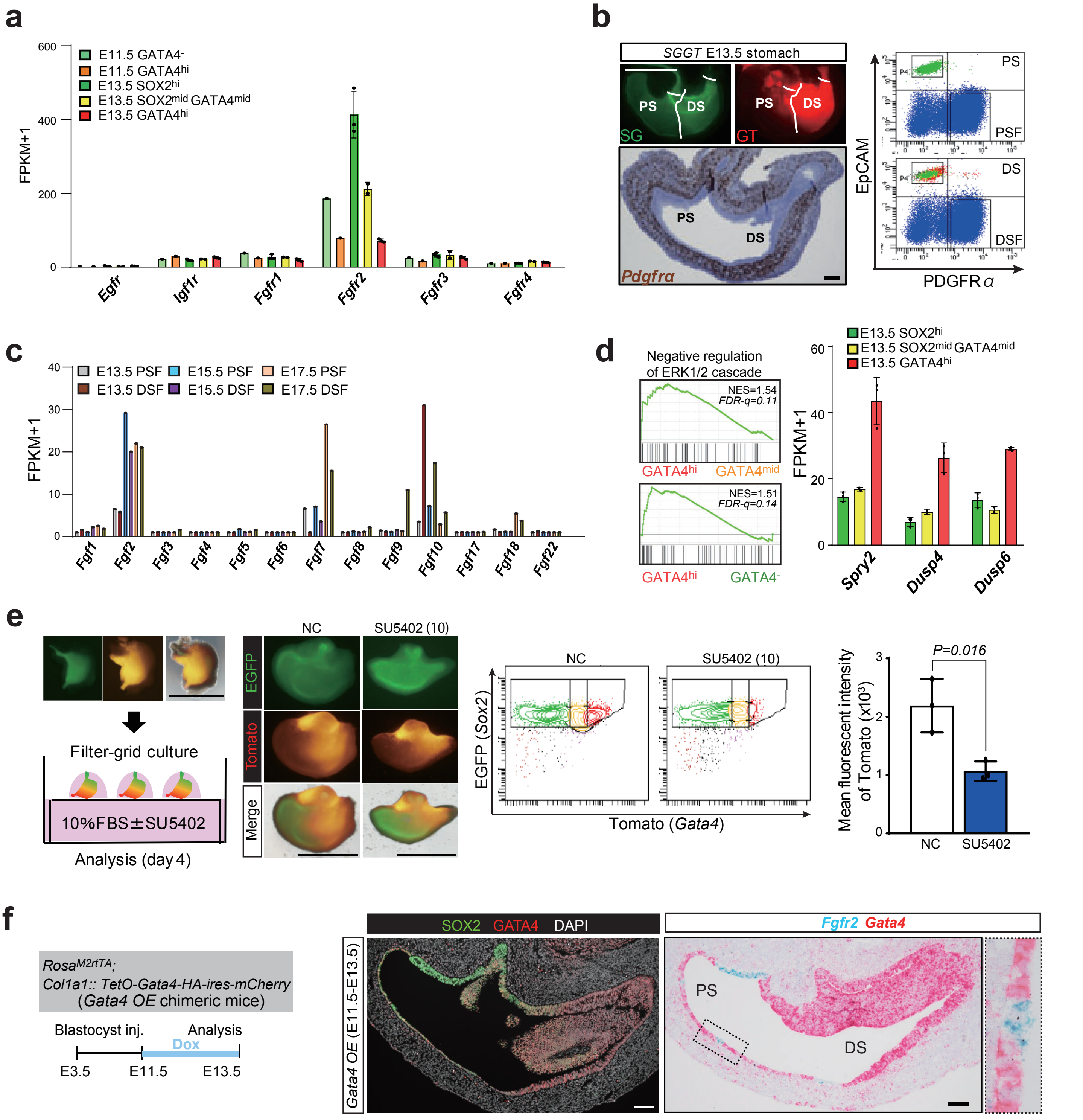

Supplementary Figure 6; Coordinated interplay between a concentration gradient of *Fgf10/Fgfr2* axis and epithelial expressions of *Gata4/Sox2*

(a) RNA-seq analyses of representative RTKs for *Sox2*$^{+}$ *Gata4*$^{-}$ (n=1) and *Sox2*$^{+}$ *Gata4*^{hi} (n=1) epithelial cells of the embryonic stomach at E11.5 and *Sox2*^{hi} (n=3), *Sox2*^{mid}*Gata4*^{mid} (n=2), and *Gata4*^{hi} (n=3) epithelial cells of the embryonic stomach at E13.5.

(b) Isolation of the stomach fibroblasts using FACS with PDGFR α-staining. PSF, proximal stomach fibroblast; DSF, distal stomach fibroblast.

(c) RNA-seq analyses of *Fgf* ligands for the PSF (n=1) and DSF (n=1) at E13.5, E15.5, and E17.5.

(d) Left: GSEA analyses of negative regulation of the ERK1/2 cascade for *Sox2*^{hi} (n=3), *Sox2*^{mid}*Gata4*^{mid} (n=2), and *Gata4*^{hi} (n=3) cells at E13.5. Right: RNA-seq analyses of *Spry2*, *Dusp4*, and *Dusp6* for *Sox2*^{hi} (n=3), *Sox2*^{mid}*Gata4*^{mid} (n=2), and *Gata4*^{hi} (n=3) cells of the *SGGT* stomach at E13.5.

(e) Left: a scheme for the explant culture experiment of *SGGT* stomachs at E11.5 for 4 days with or without SU5402 (10nM). Middle to Right: Fluorescent images, FACS analyses and the mean fluorescent intensity of td-Tomato of *SGGT* stomachs are shown. n=3 independent experiments. Data are presented as mean values ± SD. two-sided *t*-test.

(f) Left: A scheme for the *Gata4* OE experiment using *KH2-Gata4* chimeric embryos. Scale bar, 100 µm. Middle: IF analyses of SOX2 and GATA4 for the stomachs of *Gata4 OE* (*E11.5*) chimeric embryos at E13.5. Right: RNA-ISH analyses of *Fgfr2* and *Gata4* for *Gata4* OE stomachs at E13.5. Presented data are a representative image of n=3. Scale bar, 100 µm.

Supplementary Figure 7

a

Sox2-CreERT2 knock-in mESC

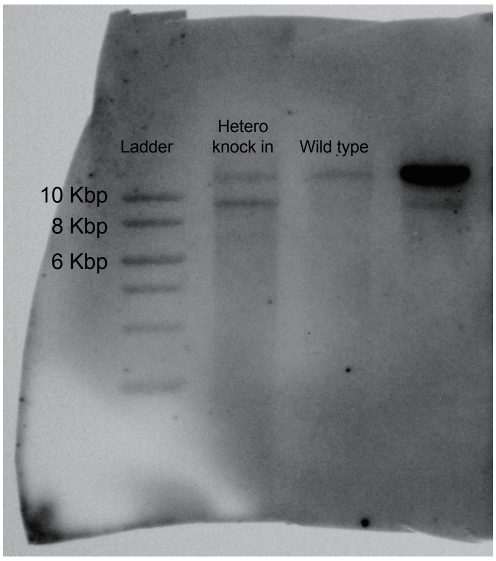

b

Gata4-CreERT2 knock-in mESC

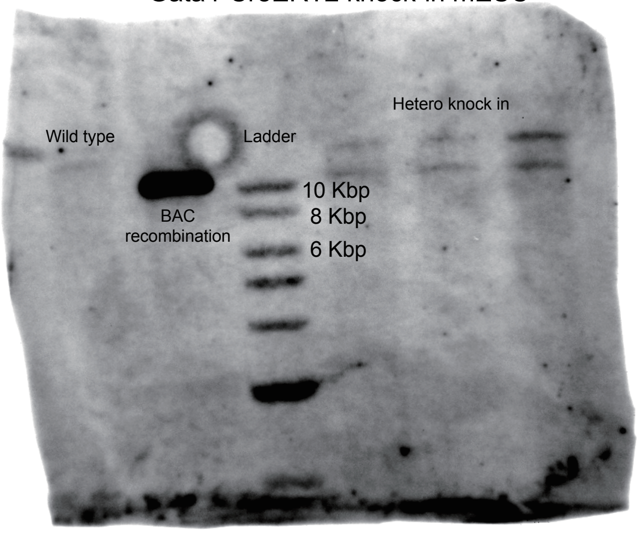

c

Gata4-tdTomato knock-in mESC

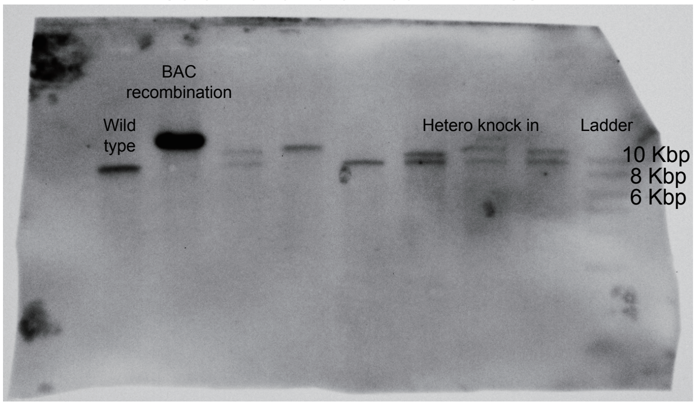

Supplementary Figure 7; Uncropped Southern blots

(a-c) Confirmation of homologous recombination at the targeted loci by Southern blots.

Supplementary Table1 : Primers used in this study

Genotyping primers

Gata4-CreERT2	Fw	ATTTTGAGCGAGTTGGGCCT
	Rv1	GTTGCATCGACCGGTAATGC
	Rv2	AGTAGTCCCCGGGAAAGAGAA
Rosa LSL-LacZ	Fw	AAAGTCGCTCTGAGTTGTTAT
	Rv1	GGAGCGGGAGAAATGGATATG
	Rv2	GCGAAGAGTTTGTCCTCAACC
Sox2-CreERT2	Fw	CATCCCAATTGCACTTCGCC
	Rv1	GTTGCATCGACCGGTAATGC
	Rv2	AATCCGGGTGCTCCTTCATG
Gata4 floxed	Fw	CCCAGTAAAGAAGTCAGCACAAGGAAAC
	Rv	AGACTATTGATCCCGGAGTGAACATT
Sox2 floxed	Fw	TGGAATCAGGCTGCCGAGAATCC
	Rv	TCGTTCTGGCAACAAGTGCTAAAGC
Δ Np63-Cre	Fw	AGTTGATGGATTGGACAG
	Rv1	GATAATCGCGAACATCTCAGG
	Rv2	AGCAGGGTCTTCTCTACTT
Sox2-EGFP	Fw1	ACCAGCTCGCAGACCTACAT
	Fw2	AAGTTCATCTGCACCACCG
	Rv1	CGGGGAGGTACATGCTGAT
	Rv2	TCCTTGAAGAAGATGGTGCG
Gata4-tdTomato	Fw	ATTTTGAGCGAGTTGGGCCT
	Rv1	GCCATGTTGTTGTCCTCGGA
	Rv2	AGTAGTCCCCGGGAAAGAGAA

qRT-PCR primers

Sox2	Fw	CATGAGAGCAAGTACTGGCAAG
	Rv	CCAACGATATCAACCTGCATGG
p63	Fw	TGCCATGCCTGTCTACAAG
	Rv	GCTGTTCCCTTCTACTCGAATC
Gata4	Fw	TTCCTCTCCCAGGAACATCAAA
	Rv	GCTGCACAACTGGGCTCTACTT
Krt7	Fw	GCCTACACGAACAAGGTGGA
	Rv	CCATGGACAGCACCACAGAT
Krt14	Fw	TGAAGACAAGGCTGGAGCAG
	Rv	ATGACCTTGGTGCGGATCTG
Lor	Fw	AACGGAGACAACAGAGCTGG
	Rv	CAATGGCTTCTTCTGGGGGA
Irx3	Fw	ATAAGACCAGAGCAGCGTCC
	Rv	GTGCCTTGGAAGTGGAGAAA
Pdx1	Fw	GAAATCCACCAAAGCTCACG
	Rv	ACGGGTCCTCTTGTTTTCCT
Fgfr2	Fw	TGCACGCAGGATGGACCTCTCT
	Rv	TGCTCCTCGGGGACACGGTTAA

Probes for Southern blot

Sox2 allele	Fw	TCAAGCCCTGGTTTTGTTTT
	Rv	CAGGAAGAGGCGTATCCAAA
Gata4 allele	Fw	AATGAGAAAGGCAAAGGCGCC
	Rv	TTTACACACCATTGCACCGCTC

Supplementary Table 2 : Antibodies and probes used in this study

Immunostaining Antibodies

**Primary Antibodies**	**SOURCE**	**IDENTIFIER**	**Dilution**
Mouse monoclonal anti-P63	Abcam	Cat#ab735	×200
Rabbit polyclonal anti-LOR	Biolegend	Cat#905101	×1000
Mouse monoclonal anti-KRT7	Abcam	Cat#ab9021	×100
Rabbit polyclonal anti-KRT14	Biolegend	Cat#19053	×1000
Rabbit monoclonal anti-CLDN18	Abcam	Cat#ab203563	×500
Goat polyclonal anti-GATA4	Santa Cruz	Cat#sc-1237	×100
Mouse monoclonal anti-GATA4	Santa Cruz	Cat#sc-25310	×100
Rabbit polyclonal anti-SOX2	Millipore	Cat#ab5603	×500
Goat polyclonal anti-SOX2	Santa Cruz	Cat#sc-17320	×100
Rabbit monoclonal anti-HA tag	CST	Cat#3724	×200
Goat polyclonal anti-PDX1	R&D	Cat#AF2419	x200
Rabbit monoclonal anti-Phospho-p44/42 Erk1/2	Cell Signaling	Cat#4376	×200
Mouse monoclonal anti-Ctnnb1	BD Biosciences	Cat#610154	×500
Rabbit monoclonal anti-Ki67	Nichirei Biosciences	Cat#418071	×1
**Secondary Antibodies, etc**	**SOURCE**	**IDENTIFIER**	**Dilution**
Mayer's Hematoxylin Solution	Wako	Cat#131-09665	×1
0.1% Eosin Y, Ethanol Solution	Wako	Cat#054-06505	×1
Contrast Red Solution	Seracare	Cat#5540-0001	×1
5-Bromo-4-chloro-3-indolyl-β-D-galactoside	Nacalai	Cat#05627-86	x20
Donkey anti-Mouse IgG (H+L) Highly Cross-Adsorbed Secondary Antibody, Alexa Fluor 488	Invitrogen	Cat#A-21202	×500
Donkey Anti-Rabbit IgG Antibody, Cy3 conjugate, Species Adsorbed	Millipore	Cat#AP182C	×500
Donkey Anti-Goat IgG, Alexa Fluor® 647 Conjugate, Species Adsorbed: H, M, R, Ch, Gp, Eq, Ht Antibody	Millipore	Cat#AP180SA6	×500
DAPI	Invitrogen	Cat#D1306	×500

FACS Antibodies

**Antibodies**	**SOURCE**	**IDENTIFIER**	**Dilution**
APC anti-mouse CD326 (Ep-CAM) Antibody	Biolegend	Cat#118213	×1000
BV421 Rat Anti-Mouse CD140A	BD	Cat#562774	×200

RNA scope probes

**Probes**	**SOURCE**	**IDENTIFIER**
Mm-*Gata4* -C2	ACD	Cat#417881-C2
Mm-*Fgfr2* -no-XHs	ACD	Cat#443501
Mm-*Fgf10*	ACD	Cat#446371
Mm-*Pdgfrα*	ACD	Cat#480661
Mm-*Cckar*	ACD	Cat#313751
Mm-*Wnt7b*	ACD	Cat#401131
Mm-*Syne1*	ACD	Cat#316511
