# Supplementary material for: Epithelial expression of Gata4 and Sox2 regulates specification of the squamous–columnar junction via MAPK/ERK signaling in mice
Source: Nat Commun. 2021 Jan 25;12:560. doi: 10.1038/s41467-021-20906-0 (PMC7835245; doi:10.1038/s41467-021-20906-0)
Supplement: Supplementary file 3 — Reporting Summary [file 41467_2021_20906_MOESM3_ESM.pdf]

nature research

Corresponding author(s): Yoshiya Kawaguchi

Last updated by author(s): Dec 16, 2020

Reporting Summary

Nature Research wishes to improve the reproducibility of the work that we publish. This form provides structure for consistency and transparency in reporting. For further information on Nature Research policies, see Authors & Referees and the Editorial Policy Checklist .

Statistics

For all statistical analyses, confirm that the following items are present in the figure legend, table legend, main text, or Methods section.

n/a	Confirmed	
☐	☒	The exact sample size (*n*) for each experimental group/condition, given as a discrete number and unit of measurement
☐	☒	A statement on whether measurements were taken from distinct samples or whether the same sample was measured repeatedly
☐	☒	The statistical test(s) used AND whether they are one- or two-sided<br>*Only common tests should be described solely by name; describe more complex techniques in the Methods section.*
☐	☒	A description of all covariates tested
☐	☒	A description of any assumptions or corrections, such as tests of normality and adjustment for multiple comparisons
☐	☒	A full description of the statistical parameters including central tendency (e.g. means) or other basic estimates (e.g. regression coefficient) AND variation (e.g. standard deviation) or associated estimates of uncertainty (e.g. confidence intervals)
☐	☒	For null hypothesis testing, the test statistic (e.g. *F*, *t*, *r*) with confidence intervals, effect sizes, degrees of freedom and *P* value noted<br>*Give P values as exact values whenever suitable.*
☒	☐	For Bayesian analysis, information on the choice of priors and Markov chain Monte Carlo settings
☒	☐	For hierarchical and complex designs, identification of the appropriate level for tests and full reporting of outcomes
☒	☐	Estimates of effect sizes (e.g. Cohen's *d*, Pearson's *r*), indicating how they were calculated

Our web collection on statistics for biologists contains articles on many of the points above.

Software and code

Policy information about availability of computer code

Data collection	RNA quality was analyzed by Bioanalyzer. RNA-seq libraries were sequenced on a NextSeq500 (Illumina) as 75 bp single reads.
Data analysis	We use Graphpad Prism7 to perform statistical analyses; Carl Zeiss Zen software to analyze Images; BD FACS Aria• to collect samples and FACS Diva to analyze data.

For manuscripts utilizing custom algorithms or software that are central to the research but not yet described in published literature, software must be made available to editors/reviewers. We strongly encourage code deposition in a community repository (e.g. GitHub). See the Nature Research guidelines for submitting code & software for further information.

Data

Policy information about availability of data

All manuscripts must include a data availability statement. This statement should provide the following information, where applicable:
- Accession codes, unique identifiers, or web links for publicly available datasets
- A list of figures that have associated raw data
- A description of any restrictions on data availability

All data of RNA-seq used in this study were deposited in the Gene Expression Omnibus (GEO) under accession number GSE143217.

Field-specific reporting

Please select the one below that is the best fit for your research. If you are not sure, read the appropriate sections before making your selection.

Life sciences study design

All studies must disclose on these points even when the disclosure is negative.

Sample size	Sample size is described in methods, figures or figure legends. qRT-PCR experiments were performed in biological triplicate. We have had enough number of sequencing reads for RNA-seq.
Data exclusions	We don't have any exclusions in this study.
Replication	All experiments (except for RNA-seq) were performed at least 3 times and we were able to confirm the reproducibility of our results.
Randomization	We don't have randomization in this study.
Blinding	There are no samples used for blinding to the group allocation in this study.

Reporting for specific materials, systems and methods

We require information from authors about some types of materials, experimental systems and methods used in many studies. Here, indicate whether each material, system or method listed is relevant to your study. If you are not sure if a list item applies to your research, read the appropriate section before selecting a response.

Materials & experimental systems

n/a	Involved in the study	
☐	☒	Antibodies
☒	☐	Eukaryotic cell lines
☒	☐	Palaeontology
☐	☒	Animals and other organisms
☐	☒	Human research participants
☒	☐	Clinical data

Methods

n/a	Involved in the study	
☒	☐	ChIP-seq
☐	☒	Flow cytometry
☒	☐	MRI-based neuroimaging

Antibodies

Antibodies used	Primary antibodies used for immunostaining analysis :<br>Mouse monoclonal anti-P63 (Abcam, ab735,1:200); Rabbit polyclonal anti-LOR (Biolegend, 905101, 1:1000); Mouse monoclonal anti-KRT7 (Abcam, ab9021, 1:100); Rabbit polyclonal anti-KRT14 (Biolegend, 19053, 1:1000); Rabbit monoclonal anti-CLDN18 (Abcam, ab203563, 1:500); Goat polyclonal anti-GATA4 (Santa Cruz, sc-1237, 1:100); Mouse monoclonal anti-GATA4 (Santa Cruz, sc-25310, 1:100); Rabbit polyclonal anti-SOX2 (Millipore, ab5603, 1:500); Goat polyclonal anti-SOX2 (Santa Cruz, sc-17320, 1:100); Rabbit monoclonal anti-HA tag (CST, 3724, 1:200); Goat polyclonal anti-PDX1 (R&D, AF2419, 1:200); Rabbit monoclonal anti-Phospho-p44/42 Erk1/2 (Cell Signaling, 4376, 1:200); Mouse monoclonal anti-Ctnnb1 (BD Biosciences, 610154, 1:500); Rabbit monoclonal anti-Ki67 (Nichirei Biosciences, 418071, 1:1)<br>Secondary antibodies used for immunostaining analysis :<br>Donkey anti-Mouse IgG (H+L) Highly Cross-Adsorbed Secondary Antibody, Alexa Fluor 488 (Invitrogen, A-21202, 1:500); Donkey Anti-Rabbit IgG Antibody, Cy3 conjugate, Species Adsorbed (Millipore, AP182C, 1:500); Donkey Anti-Goat IgG, Alexa Fluor® 647 Conjugate, Species Adsorbed: H, M, R, Ch, Gp, Eq, Ht Antibody (Millipore, AP180SA6, 1:500) ;DAPI (Invitrogen, D1306, 1:500).<br>Antibodies used for FACS analysis:<br>APC anti-mouse CD326 (Ep-CAM) (Antibody Biolegend, 118213, 1:1000); BV421 Rat Anti-Mouse CD140A (BD, 562774, 1:200).
Validation	The staining were performed according to the manufacture's protocol, and the antibodies were validated using positive controls and negative controls (IgG isotype).

Animals and other organisms

Policy information about studies involving animals; ARRIVE guidelines recommended for reporting animal research

Laboratory animals	We use Sox2-CreERT2, Gata4-CreERT2, ə Np63-Cre, R26R, Sox2-EGFP, Gata4-tdTomato, Sox2 flox, Gata4 flox, and KH2-Gata4 mouse strains. The mice were maintained on a genetic background of mouse cell lines mixed between 129X1/SvJ and C57BL/6.
Wild animals	Pregnant C57BL/6 females carrying embryos (E9.5, E11.5, E13.5, E15.5, E17.5, E18.5) were obtained from SLC. Pseudopregnant ICR and male and female ICR (8-10 week old) were obtained from SLC to make chimeric animals.

Field-collected samples	N/A
Ethics oversight	All experiments using animals were performed under the ethical guidelines of Kyoto University and University of Tokyo.

Note that full information on the approval of the study protocol must also be provided in the manuscript.

Human research participants

Policy information about studies involving human research participants

Population characteristics	Human esophageal-gastric junction and uterine cervix were provided by the Kyoto university department of surgery and gynecology. Slides were provided by the department of pathology. Human samples are included both male and female. Human patients include normal and Barrett's esophagus. The age of the patients are from 30-70.
Recruitment	*Describe how participants were recruited. Outline any potential self-selection bias or other biases that may be present and how these are likely to impact results.*
Ethics oversight	Patients-derived paraffin-embedded tissue samples were used in accordance with ethical guidelines in Kyoto University Hospital.

Note that full information on the approval of the study protocol must also be provided in the manuscript.

Flow Cytometry

Plots

Confirm that:

- [x] The axis labels state the marker and fluorochrome used (e.g. CD4-FITC).
- [x] The axis scales are clearly visible. Include numbers along axes only for bottom left plot of group (a 'group' is an analysis of identical markers).
- [x] All plots are contour plots with outliers or pseudocolor plots.
- [x] A numerical value for number of cells or percentage (with statistics) is provided.

Methodology

Sample preparation	Embryonic mouse stomachs were incubated with Accumax and digested into single cells.
Instrument	We use BD FACS Aria II to collect data.
Software	We use BD FACS Diva to analyze data.
Cell population abundance	90% of the dispersed cells were single cells and around 10% of the single cells were EpCAM+ epithelial cells. 95% of EpCAM+ epithelial cells were Sox2-EGFP+ cells.
Gating strategy	Single cells were first gated by FSC-A vs SSC-A, FSC-W vs FSC-H, and SSC-W vs SSC-H. The single cells were further divided into EpCAM+ epithelial cells or PDFGRa+ mesenchymal cells by surface marker staining. Gating strategies to determine Sox2-EGFP and Gata4-tdTomato expression levels in the single epithelial cells were described in Extended Data Fig. 5a.

- [x] Tick this box to confirm that a figure exemplifying the gating strategy is provided in the Supplementary Information.
